# Exploring the Link Between Obstructive Sleep Apnea and Neuropsychiatric Disorders: Role of Neuroinflammatory Mechanisms

**DOI:** 10.7759/cureus.112096

**Published:** 2026-07-05

**Authors:** Mohammad Raza Mehdi, Zahraa Munaf Shakir Al-Qassab, Omar Sabuni, Mriganka Rai, Tirath Patel, Rishil Patel, Laraib Iftikhar

**Affiliations:** 1 Emergency Medicine, Hull Royal Infirmary, Hull, GBR; 2 Internal Medicine, Metropolitan Hospital Center, New York, USA; 3 Medicine, Hackensack Meridian Health Palisades Medical Center, New Jersey, USA; 4 Internal Medicine, Medical University Sofia, Sofia, BGR; 5 Medicine, Trinity School of Medicine, Kingstown, VCT; 6 Medicine, Faculty of Medicine, St. Martinus University, Willemstad, CUW; 7 Medicine, California Institute of Behavioral Neurosciences and Psychology, California, USA

**Keywords:** anxiety, blood-brain barrier (bbb), cognitive impairment and dementia, continuous positive airway pressure (cpap), depression, depression in chronic illness, neuro-psychiatric, obstructive sleep apnea syndrome (osas), sleep disorderd breathing, sleep fragmentation

## Abstract

Obstructive sleep apnea syndrome (OSAS) is a prevalent sleep-related breathing disorder characterized by recurrent episodes of upper airway obstruction during sleep, resulting in intermittent reductions or complete cessation of airflow. These interruptions reduce oxygen saturation and disrupt normal sleep architecture, frequently resulting in daytime fatigue and adverse health outcomes. Recent research has provided increasing evidence that OSAS may be associated with neuroinflammation, defined as inflammation within the brain and nervous system. Such neuroinflammation may contribute to the development of conditions including depression, anxiety, Alzheimer’s disease, and Parkinson’s disease.

This narrative review examines the association between OSAS and neuroinflammation and outlines the potential biological mechanisms involved. Intermittent hypoxemia and recurrent sleep fragmentation are thought to promote oxidative stress, neuroinflammation, and neuronal injury, ultimately contributing to impaired memory, executive function, emotional regulation, and overall neurological function. Over time, these processes can impair memory, executive function, emotional regulation, and overall neurological function. The review also highlights key risk factors and clinical manifestations and emphasizes the importance of early diagnosis and intervention for OSAS. Evidence from both experimental animal studies and human clinical studies is discussed to highlight current understanding while distinguishing established findings from emerging hypotheses.

In summary, this review indicates that neuroinflammation may represent an important mechanistic pathway linking OSAS with depression, anxiety, cognitive impairment, and neurodegenerative disorders. However, much of the available evidence remains associative, and further longitudinal and biomarker-driven studies are required to clarify causal relationships and determine the long-term impact of interventions such as continuous positive airway pressure (CPAP) therapy. Improved understanding of these mechanisms may facilitate earlier diagnosis, risk stratification, and the development of targeted therapeutic strategies for individuals with OSAS.

## Introduction and background

Sleep is a fundamental biological process that is essential for maintaining physical health, cognitive performance, and psychological well-being. Increasing evidence suggests that sleep disorders are associated with a range of neurological and psychiatric conditions, emphasizing the importance of understanding their underlying mechanisms [1,2]. One extensively studied sleep disorder is obstructive sleep apnea syndrome (OSAS). It is diagnosed primarily by overnight polysomnography and is commonly defined by an apnea-hypopnea index (AHI) of ≥5 events/hour in symptomatic individuals or ≥15 events/hour irrespective of symptoms. The disorder typically presents with loud snoring, witnessed apneic episodes, nocturnal gasping or choking, excessive daytime sleepiness, morning headaches, impaired concentration, and reduced quality of life. OSAS disrupts the sleep cycle that consists of three main stages: N1 and N2, known as light sleep; N3, called deep sleep; and rapid eye movement (REM) sleep, which is also known as vivid dreaming, crucial for physical restoration, memory consolidation, and cognitive function [3]. OSAS disrupts normal sleep architecture through recurrent arousals, reducing both REM sleep continuity and slow-wave (N3) sleep, thereby impairing restorative sleep and cognitive recovery [4]. OSAS is characterized by recurrent upper airway obstruction during sleep, resulting in intermittent hypoxia (IH), recurrent arousals, and sleep fragmentation (SF), which have been associated with an increased risk of cardiovascular and neurodegenerative disorders [5,6,7,8].

Obstructive sleep apnea syndrome is one of the most common sleep-related breathing disorders and represents a significant global public health concern. Epidemiological studies suggest that its prevalence increases with age, obesity, and male sex, while a substantial proportion of affected individuals remain undiagnosed worldwide [1]. Although OSAS may occur at any age, it is most prevalent among middle-aged and older adults, while children and younger adults may also be affected, particularly in the presence of adenotonsillar hypertrophy, craniofacial abnormalities, or obesity [1]. There is also widespread evidence of excessive daytime sleepiness, unstable interpersonal relationships, increased road traffic accidents, and reduced productivity at work and impaired academic performance in children and adolescents, resulting in poor quality of life for patients [9,10]. Neuropsychiatric comorbidities are also common among individuals with OSAS. Previous studies have consistently reported a higher prevalence of depression, anxiety, and cognitive impairment in patients with OSAS than in the general population [10,11]. Collectively, these observations provide a compelling rationale for exploring how OSAS may contribute to neuropsychiatric disorders through neuroinflammatory mechanisms. Recent research in sleep medicine and neuroimmunology suggests that the neurological consequences of OSAS extend beyond sleep disruption alone. Recurrent intermittent hypoxia and sleep fragmentation are associated with oxidative stress, systemic inflammation, and vascular dysfunction [11,12,13]. Higher levels of inflammatory biomarkers, including interleukin-6 (IL-6), tumor necrosis factor-α (TNF-α), and C-reactive protein (CRP), have been consistently reported in patients with OSAS. These inflammatory processes may contribute to blood-brain barrier (BBB) dysfunction, activation of microglia and astrocytes, and neuronal injury, providing a potential mechanistic link between OSAS and cognitive and emotional dysfunction [13]. Collectively, these findings suggest that neuroinflammation may represent an important mechanistic link between OSAS and long-term cognitive and emotional dysfunction [8,9,11,12,13].

Neuroinflammation has emerged as a potential mechanism linking OSAS with cognitive decline and mood disturbances. The evolving changes in sleep cycles and quality in patients with OSAS, along with altered cerebral blood flow and neurotransmitter activity, contribute to impaired neuroplasticity, vaso-neural alterations, and disruption of cellular redox reactions. This then leads to oxidative stress through repeated nocturnal hypoxemia and SF, which remain under scrutiny [12,13,14]. This neural injury can lead to a range of dysfunctions, including emotional changes, cognitive decline, loss of sensation, and motor disturbances [14,15,16]. In addition, these pathological processes are implicated in the development of neurodegenerative disorders such as Alzheimer’s disease (AD) and Parkinson’s disease (PD) [3,17,18,19]. Accumulating evidence suggests that intermittent hypoxia may contribute to neuroinflammatory and neurodegenerative processes through mechanisms including oxidative stress, blood-brain barrier dysfunction, glial activation, and inflammatory cytokine release. Although these mechanisms are increasingly recognized, the extent to which they contribute to long-term neuropsychiatric and neurodegenerative outcomes remains an area of ongoing investigation and is explored in greater detail throughout this review [12,20,21].

The aim of this narrative review is to critically evaluate the current evidence regarding the association between OSAS and neuropsychiatric disorders, with particular emphasis on cognitive impairment, mood disorders, and the potential role of neuroinflammatory mechanisms. In the background of IH and SF, OSAS is an extensively recognized risk factor for cognitive decline, cerebrovascular disease, and other disorders [6,7,15]. The key mechanisms underlying this relationship between OSAS and neural insults remain unclear; therefore, the relationship between OSAS and neuropsychiatric disorders is assayed in this review.

## Review

Methods

Literature Search and Study Selection

This narrative review was conducted to summarise the current evidence regarding the association between obstructive sleep apnoea syndrome (OSAS), neuroinflammation, and neuropsychiatric disorders. A literature search was performed using PubMed/MEDLINE, Scopus, and Google Scholar to identify relevant peer-reviewed articles published in English from database inception to March 2026. Reference lists of relevant review articles were also screened manually to identify additional eligible studies. Search terms and Boolean operators included combinations of "obstructive sleep apnoea" OR "sleep-disordered breathing", "intermittent hypoxia", "sleep fragmentation", "neuroinflammation", "cognitive impairment", "depression", "anxiety", "Alzheimer's disease", "Parkinson's disease", "microglia", "blood-brain barrier", and "continuous positive airway pressure (CPAP)".

Study selection was guided by predefined eligibility criteria developed to address the objectives of this narrative review. The population included adults with OSAS and relevant experimental animal models investigating intermittent hypoxia, sleep fragmentation, or neuroinflammatory mechanisms. The exposures or interventions of interest were OSAS, intermittent hypoxia, sleep fragmentation, and CPAP therapy. Outcomes included neuroinflammatory biomarkers, blood-brain barrier dysfunction, oxidative stress, cognitive impairment, mood disorders, neurodegenerative changes, and treatment-related neurological outcomes. Original research articles, systematic reviews, meta-analyses, narrative reviews, and experimental studies relevant to the objectives of this review were included. Conference abstracts, editorials, case reports, non-English publications, duplicate studies, and articles unrelated to neuroinflammatory or neuropsychiatric mechanisms in OSAS were excluded. Where multiple publications addressed similar topics, priority was given to the most recent systematic reviews, meta-analyses, and high-quality original studies.

As this was a narrative review, no formal assessment of study quality or risk of bias was undertaken. Instead, studies were selected for their relevance to the review's objectives and their contribution to understanding the relationship among OSAS, neuroinflammation, and neuropsychiatric disorders.

As this study was conducted as a narrative review, the identified literature was synthesized qualitatively rather than quantitatively. Evidence from experimental animal studies, observational clinical studies, systematic reviews, meta-analyses, and narrative reviews was critically evaluated and integrated to provide a comprehensive overview of the current understanding of the neuroinflammatory mechanisms linking obstructive sleep apnoea syndrome (OSAS) with neuropsychiatric dysfunction. Greater emphasis was placed on recent peer-reviewed evidence, while landmark studies were retained to provide historical context where appropriate. Owing to the narrative design of this review, a formal Preferred Reporting Items for Systematic Reviews and Meta-Analyses (PRISMA)-guided study selection process and quantitative risk-of-bias assessment were not performed.

OSAS overview

Obstructive sleep apnoea syndrome results from upper airway collapse during sleep, leading to repeated arousals and desaturation [22]. Reduced pharyngeal dilator tone during sleep contributes to airway collapse, occurring more frequently in the rapid eye movement (REM) stage of the sleep cycle [22]. In addition to male sex, recognized risk factors for OSAS include advancing age, family history, obesity, increased neck circumference, craniofacial abnormalities, smoking, alcohol consumption, glucose intolerance, hyperlipidemia, and other cardiometabolic disorders. These factors not only increase the risk of OSAS but may also contribute to neuropsychiatric dysfunction through shared inflammatory and vascular mechanisms [11,17,22,23]. Intermittent hypoxia (IH) and sleep fragmentation (SF) are noticeable factors that instigate neurodegenerative (ND) decline, such as cognitive impairment and executive dysfunction.

Intermittent Hypoxia Channelling between OSAS and Neuro-Inflammation

According to studies, intermittent hypoxia (IH) causes an increase in oxidative stress and neuronal apoptosis that damages the central nervous system (CNS), leading to neurological decline [7,20,21,24]. Experimental animal studies suggest that IH may promote neuronal apoptosis by damaging structures within the hippocampus and frontal cortex [23]. Studies demonstrated that IH-induced spatial learning decline peaked on days 1 and 2, then slowed thereafter [25]. Another study showed that when exposed to 48 hours of IH, rats at post-natal age between days 10-25 showed significant apoptosis, signalling that IH can lead to cell apoptosis in those CNS regions and hence could potentially be one of the causes of brain damage in OSAS [17,26]. Increased oxidative stress with IH, due to the augmentation of reactive oxygen species (ROS) and the activation of nicotinamide adenine dinucleotide phosphate (NADPH)oxidase, has been shown to potentially contribute to neuronal injury [27,28]. IH also disrupts the function and structure of neuronal synapses by activating astrocytes, thereby contributing to neuronal injury [25]. Finally, injury to endothelial nitric oxide synthase (NOS), leading to reduced nitric oxide (NO) production, impairs blood flow and causes vascular damage, further harming neuronal mechanisms [8,21].

Experimental and clinical evidence suggests that intermittent hypoxia initiates a series of cellular and molecular events that contribute to neuroinflammation [20,25,28]. In OSAS, repeated cycles of hypoxia and reoxygenation lead to the formation of reactive oxygen species (ROS) and activate NADPH oxidases, causing oxidative stress [25,27,28]. This redox imbalance activates pathways that promote inflammation [20,25]. For example, ROS signals stimulate nuclear factor kappa B (NF-κB) and hypoxia-inducible factor-1 alpha​​​​​​​ (HIF-1α), which in turn promote the production of pro-inflammatory cytokines such as TNF-α, IL-6, and IL-1β [13,20]. These factors induce chemokine and cytokine production, activating endothelial and peripheral immune cells. Together, these processes set the stage for inflammation and can directly impact the CNS [16,20].

The core association driving brain inflammation is microglial activation, which is induced by chronic intermittent hypoxia (CIH) [25,28]. CIH causes structural changes in microglia, leading to enhanced NF-κB signalling, increased inducible nitric oxide synthase (iNOS) expression, and the release of inflammatory factors, including IL-1β and IL-18 [28]. These processes lead to the release of chemical messengers that bind to the cell itself and neighbouring cells, thereby playing a major role in neuronal damage [25,29].

Emerging evidence suggests that blood-brain barrier (BBB) dysfunction represents an important mechanistic link between intermittent hypoxia and neuroinflammation in OSAS. Experimental evidence suggests that repeated cycles of hypoxia and re-oxygenation may compromise endothelial tight junction integrity, elevating BBB permeability and enabling peripheral inflammatory mediators to infiltrate the central nervous system [16,30]. Studies in mice have shown that chronic intermittent hypoxia can cause early BBB disruption, which then heightens oxidative stress, activates microglia, and makes neurons more vulnerable [16]. These changes can further disturb neuronal balance and may speed up neurodegenerative processes, especially in conditions like Alzheimer’s disease that involve cognitive decline [18,30,31].

Peripheral inflammation, characterized by elevated pro-inflammatory cytokines, can amplify BBB permeability. Cumulative studies suggest that CIH also promotes synaptic loss and neuronal apoptosis and disrupts tau phosphorylation and amyloid precursor protein - changes also observed in the pathology of Alzheimer’s disease (AD) [18,19]. The joint insult caused by mitochondrial dysfunction, oxidative stress, and cytokines leads to defective synaptic plasticity and cognitive decline observed in OSAS [20,32].

Overall, IH, as an influential catalyst, enhances these processes, leading to neuronal injury, BBB disruption, and microglial activation, providing a biological foundation for increased susceptibility to neurodegeneration in OSAS [28]. A better understanding of these pathophysiological mechanisms may facilitate the identification of novel therapeutic targets, including antioxidant strategies and interventions such as continuous positive airway pressure (CPAP), which may help restore endothelial function, attenuate neuroinflammation, and preserve cognitive function [1]. In summary, IH promotes oxidative stress, endothelial dysfunction, and microglial activation, together forming a biological bridge between OSAS and neurodegeneration [8].

Sleep Fragmentation: Cause for Neuroinflammation in OSAS

Alongside intermittent hypoxia (IH), recurrent sleep fragmentation (SF) represents one of the principal drivers of neuroinflammation in obstructive sleep apnoea syndrome (OSAS) [33]. The architecture and continuity of restorative sleep stages, particularly slow-wave and REM sleep, are disrupted by frequent arousals. Recurrent respiratory events disrupt the normal architecture and continuity of sleep by increasing cortical arousals and reducing the duration of restorative sleep stages, particularly slow-wave sleep (N3) and rapid eye movement (REM) sleep. Slow-wave sleep is essential for synaptic homeostasis, memory consolidation, glymphatic clearance, and metabolic restoration, whereas REM sleep plays a critical role in emotional regulation, learning, and cognitive processing. Consequently, repeated disruption of these sleep stages may impair neuronal repair mechanisms, alter cerebral blood flow and neurotransmitter regulation, and promote oxidative stress and inflammatory signalling. These alterations are thought to contribute to glial activation, synaptic dysfunction, and impaired neuroplasticity, providing a mechanistic link between disturbed sleep architecture and the neuropsychiatric manifestations observed in patients with OSAS [33,34,35]. This chronic disruption plays a pivotal role in the neuroinflammatory process by disturbing homeostasis, oxidative balance, glial activity, and synaptic stability [20,22].

**Figure 1:**
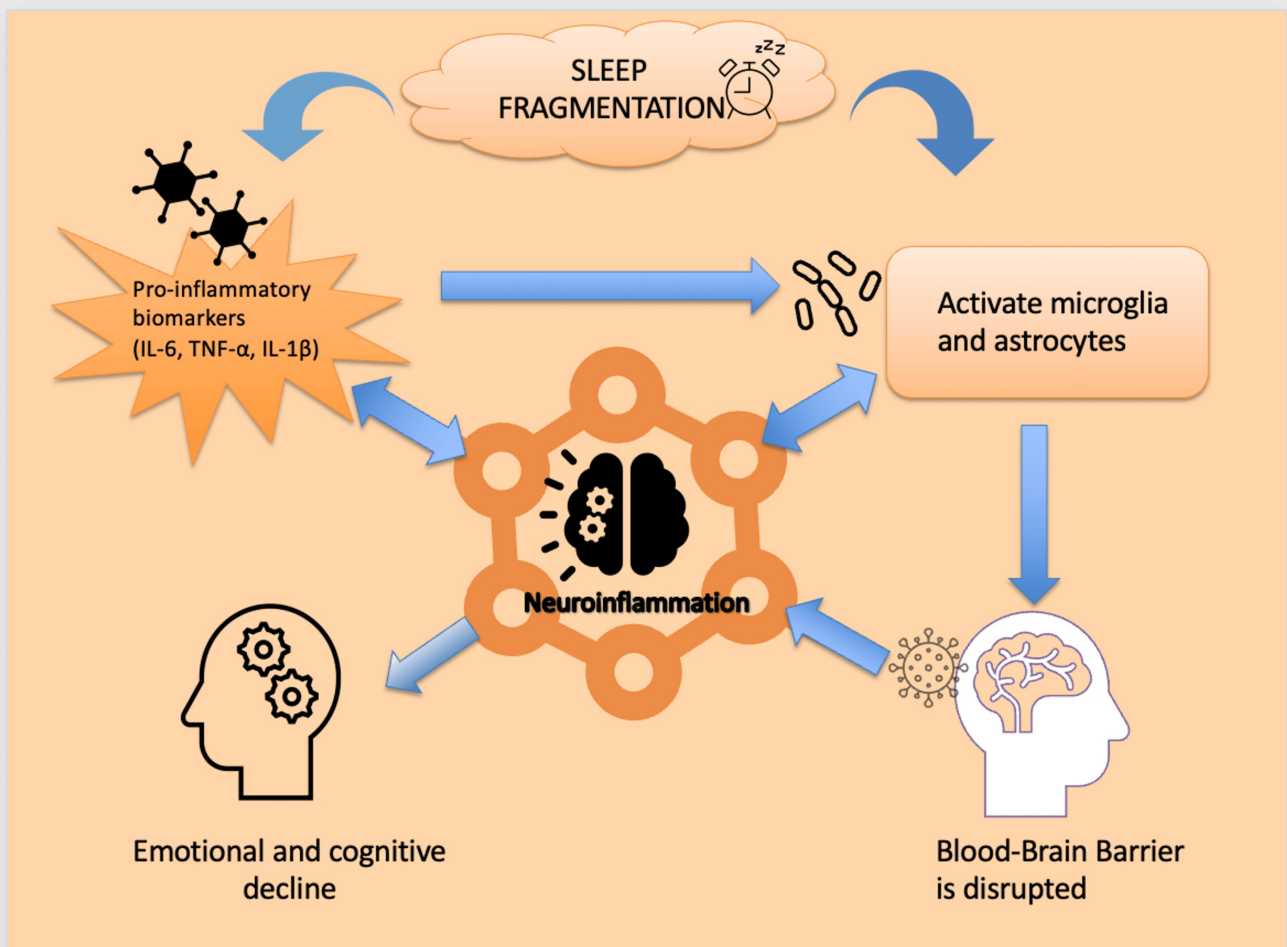
Neuroinflammatory Mechanisms of Sleep Fragmentation in OSAS The diagram illustrates how sleep fragmentation in obstructive sleep apnea (OSAS) triggers elevations in pro-inflammatory biomarkers (IL-6, TNF-α, IL-1β), driving microglial and astrocytic activation and contributing to blood–brain barrier disruption. These converging pathways propagate central neuroinflammation, resulting in cognitive and emotional decline. Bidirectional arrows represent the self-reinforcing cycle in which neuroinflammation further amplifies cytokine release and glial reactivity, exacerbating sleep disruption and perpetuating the inflammatory loop. IL: interleukin; TNF-α: tumor necrosis factor alpha This is an original image created by M Raza Mehdi on PowerPoint (Microsoft Corporation, Redmond, USA). No AI features were used.

Accumulating evidence demonstrates increased production of inflammatory mediators, including IL-6, TNF-α, and IL-1β, in association with recurrent arousals. These inflammatory responses are thought to contribute to hyperactivation of the sympathetic nervous system and the hypothalamic-pituitary-adrenal (HPA) axis [13,34]. These mediators signal through the BBB, inducing microglial and astrocyte activation [30]. Emerging evidence suggests that SF plays a bidirectional role in neuroinflammation. Recurrent arousals initiate inflammatory signalling through increased oxidative stress, activation of NF-κB, and release of pro-inflammatory cytokines, including IL-6, TNF-α, and IL-1β. In turn, these inflammatory mediators further disrupt normal sleep architecture by impairing slow-wave and REM sleep, increasing arousal frequency, and altering central sleep-wake regulatory pathways. This reciprocal interaction creates a self-perpetuating cycle in which sleep fragmentation amplifies neuroinflammation, while neuroinflammation further worsens sleep disruption [34,35]. Experimental studies in mice have shown that induced SF increases cortical and hippocampal cytokine expression, enhances NF-κB activity, and thereby promotes neuroinflammatory signalling [34]. The proposed mechanistic relationship between sleep fragmentation, inflammatory mediators, glial activation, blood-brain barrier dysfunction, and neuroinflammation is depicted in Figure 1.

Experimental evidence suggests that chronic SF primes microglia to increase ROS production and upregulate pro-inflammatory mediators, making microglia a central component of CNS inflammation [7,35]. The resulting microglial response elevates neuroimmune activity, while astrocytes release cytokines and reactive oxygen/nitrogen species that compromise synaptic homeostasis and neuronal integrity [25,28]. The combined actions of cytokines and glial activation form an inflammatory matrix that contributes to neural insults, cognitive decline, emotional instability, and autonomic dysregulation [20,34].

In both humans and animals, loss of consolidated sleep results in neurobehavioral deficits and impaired clearance of metabolic waste, including phosphorylated tau and amyloid-β [36]. The accumulation of these neurotoxic mediators may further amplify microglial activation and neuroinflammatory responses. Consequently, SF, together with IH, is thought to perpetuate chronic neuroinflammation and contribute to the development of neurological dysfunction and neuropsychiatric manifestations [25,34]. Beyond glial activation and inflammatory signalling, impaired clearance of neurotoxic metabolites has recently emerged as an additional mechanism linking sleep fragmentation with neurodegeneration.

Emerging evidence suggests that additional mechanisms, including glymphatic dysfunction, may also contribute to the neurological consequences of sleep fragmentation in OSAS. Recurrent sleep disruption in OSAS may impair glymphatic clearance, leading to the accumulation of these pathological proteins and intensifying neuroinflammatory responses [3,37]. Experimental and clinical studies increasingly support the hypothesis that disrupted sleep architecture promotes amyloid deposition and tau pathology, which may accelerate neurodegenerative disorders such as Alzheimer’s disease [19,36,37].

Consequently, sleep fragmentation may not only perpetuate neuroinflammation but also contribute to long-term neuronal vulnerability and cognitive deterioration. Overall, SF may contribute to a state of chronic neural stress. Together, IH and SF promote inflammatory and oxidative insults, heightening the risk of cognitive decline and neurodegenerative pathology in patients with untreated OSAS [34].

The principal studies evaluating the association between OSAS, neuroinflammation, and neuropsychiatric outcomes are summarised in Table 1. Collectively, these studies provide evidence from experimental models, clinical investigations, systematic reviews, and meta-analyses supporting the mechanistic pathways discussed below.

**Table 1:** Summary of key clinical and experimental studies evaluating the relationship between obstructive sleep apnoea syndrome (OSAS), neuroinflammation, and neuropsychiatric outcomes. OSAS: obstructive sleep apnoea syndrome; NF-κB: nuclear factor kappa B; TNF-α: tumor necrosis factor alpha; MAPK: mitogen-activated protein kinase; BBB: blood-brain barrier; CIH: chronic intermittent hypoxia; CPAP: continuous positive airway pressure; fMRI: functional magnetic resonance imaging

Author	Study Type	Year	Country	Population/Experimental Model	Focus of Study	Key Outcomes
Lam et al. [1]	Review	2023	Australia	Adults with dementia and sleep disorders (reviewed literature)	Sleep disorders, dementia risk, and neuroinflammatory mechanisms	Identified chronic intermittent hypoxia, sleep fragmentation, neuroinflammation, cerebrovascular dysfunction, and impaired amyloid clearance as mechanisms linking OSAS with increased dementia risk.
Mahalakshmi et al. [4]	Review	2022	India	Experimental animal and human sleep deprivation studies	Neuroinflammatory signalling pathways activated by sleep deprivation	Demonstrated activation of NF-κB, TNF-α, MAPK and inflammasome pathways leading to microglial activation, oxidative stress and neuronal injury.
Rosenzweig et al. [9]	Review	2015	UK	Adults with OSAS (clinical and neuroimaging studies)	Brain structural and functional changes in OSAS	Summarised neuroimaging evidence of grey matter injury, hippocampal dysfunction, and impaired executive function associated with untreated OSAS.
Kerner & Roose [11]	Review	2016	USA	Adults with OSAS, depression, and cognitive impairment	Association between OSAS, depression, and cognitive impairment	Reviewed evidence showing intermittent hypoxia, sleep fragmentation, and inflammation contribute to depression and cognitive impairment through oxidative stress and altered neurotransmission.
Leng et al. [14]	Systematic review & meta-analysis	2017	USA	Adults with sleep-disordered breathing	Sleep-disordered breathing and cognitive impairment	Meta-analysis showed poorer executive function, attention, and memory, together with increased risk of mild cognitive impairment and dementia.
Roche et al. [16]	Experimental	2024	France	Murine intermittent hypoxia model	Blood-brain barrier dysfunction	Intermittent hypoxia increased BBB permeability, endothelial dysfunction, and neurovascular injury.
Baril et al. [18]	Review	2018	Canada	Adults with OSAS (clinical biomarker studies)	Biomarkers of dementia in OSAS	Reported alterations in amyloid-β and tau biomarkers supporting early neurodegenerative changes.
Bubu et al. [19]	Systematic review & meta-analysis	2017	USA	Adults with sleep disorders and Alzheimer's disease	Sleep disorders and Alzheimer's disease	Demonstrated association between sleep disorders and increased risk of Alzheimer's disease, cognitive decline, and dementia.
Pham et al. [22]	Review	2015	USA	Human and experimental studies on OSAS pathophysiology	Pathophysiology of OSAS	Described upper airway collapsibility, ventilatory instability, and recurrent intermittent hypoxia leading to neurological consequences.
Yang et al. [25]	Review	2013	China	Experimental and clinical microglial studies	Microglial activation in OSAS	Intermittent hypoxia activates microglia, promoting oxidative stress, cytokine release and neuronal dysfunction.
Daulatzai et al. [31]	Review	2015	USA	Adults with OSAS and cognitive dysfunction	Neurodegeneration and cognitive dysfunction	Proposed oxidative stress, BBB dysfunction, and neuroinflammation accelerate cognitive decline.
Kazim et al. [33]	Experimental	2022	USA	Mouse model of chronic intermittent hypoxia	CIH and Alzheimer's pathology	CIH accelerated amyloid-β deposition, tau propagation, hippocampal dysfunction, and memory impairment.
Gnoni et al. [34]	Narrative review	2022	UK	Experimental and clinical neuroinflammation studies	Neuroinflammatory mechanisms in OSAS	Highlighted microglial activation, BBB dysfunction, oxidative stress, and inflammatory cytokine signalling.
Kaminska et al. [45]	Review	2015	Canada	Adults with OSAS and Parkinson's disease	OSAS and Parkinson's disease	Suggested CPAP may improve cognitive and motor outcomes in patients with coexisting OSAS.
Khazaie et al. [46]	Systematic review	2017	Iran	Adults undergoing resting-state fMRI	Resting-state fMRI changes in OSAS	Demonstrated widespread functional connectivity changes correlating with attention, executive function and memory deficits.

OSAS and neuropsychiatric dysfunction: role of neuroinflammation and CPAP use

Neuroinflammation Mechanisms

Growing evidence suggests that untreated OSAS is associated with cognitive decline, mood instability, and affective disorders [8,10]. These associations are thought to be mediated by IH and SF, which promote neuroinflammation through activation of inflammatory cytokines such as IL-6, TNF-α, CRP, and IL-1β [11,13]. As mentioned above, these factors also participate in charging the microglia to cause further neuroinflammation [12,29]. Studies have consistently reported that patients with OSAS who exhibit depressive symptoms or cognitive dysfunction have elevated circulating levels of inflammatory biomarkers, particularly IL-6 and CRP, further supporting an association between OSAS, systemic inflammation, and neuropsychiatric dysfunction [12,38]. IH and SF also induce astrocytes and microglia to begin functioning alongside factors that cause neuronal inflammation, impaired synaptic function, and neurotransmitter dysfunction [19,33]. These factors include NOD-like receptor family pyrin domain-containing 3 (NLRP3) ​​​​​​​inflammasome, caspase-1, and downstream cytokines IL-1β and IL-18 [20,34]. The mechanisms mediated by these factors are commonly observed in anxiety and depression [11,38].

Another evidence suggests that glymphatic dysfunction may further explain the relationship between sleep fragmentation and cognitive dysfunction in obstructive sleep apnoea syndrome (OSAS). Because glymphatic activity is greatest during deep (slow-wave) sleep, the recurrent disruption of sleep architecture characteristic of OSAS may impair metabolic waste clearance from the brain. The glymphatic system facilitates the removal of neurotoxic metabolites, including amyloid-β and hyperphosphorylated tau proteins, and its dysfunction has been proposed as a potential mechanism linking OSAS with cognitive impairment and neurodegenerative disease [3,36,37].

Continuous Positive Airway Pressure (CPAP) and Its Use

Continuous positive airway pressure (CPAP) is the first-line treatment for moderate-to-severe OSAS. CPAP delivers a continuous stream of pressurized air through a nasal or oronasal mask during sleep, preventing upper airway collapse and maintaining airway patency. By reducing IH and SF, CPAP helps restore normal sleep architecture and oxygenation, thereby attenuating oxidative stress, systemic inflammation, and neuroinflammatory responses implicated in cognitive dysfunction and mood disorders. CPAP therapy is generally well tolerated but requires appropriate mask fitting, patient education, and good adherence to achieve optimal clinical benefit. Treatment pressure is individualized following CPAP titration or auto-adjusting CPAP devices, with the goal of eliminating obstructive respiratory events while maintaining patient comfort and adherence. Common adverse effects include nasal dryness or congestion, mask discomfort, skin irritation, aerophagia, and claustrophobia, which may reduce long-term compliance. Although CPAP is primarily used in adults with moderate-to-severe OSAS, it may also be prescribed for selected pediatric patients under specialist supervision when clinically indicated [5,10,39,40].

Depression

Depression is a common neuropsychiatric comorbidity in patients with OSAS. Growing evidence suggests that recurrent sleep fragmentation, intermittent hypoxia, and persistent neuroinflammation contribute to the development of depressive symptoms in affected individuals [10,37,38]. People suffering from depression and poor sleep in OSAS are found to have amplified serum levels of IL-6, TNF-α, and CRP [13,39]. These inflammatory mediators activate several downstream neuroinflammatory pathways, including the indoleamine 2,3-dioxygenase (IDO)-kynurenine pathway, which has been implicated in the pathogenesis of depression [37,38]. There is also strong evidence suggesting that IL-1β and TNF-α disrupt hippocampal function and synaptic plasticity, mechanisms commonly observed in major depressive disorder [11,39]. Current evidence suggests that neuroinflammatory pathways associated with OSAS may contribute to depressive symptoms through disruption of neurotransmitter homeostasis and dysregulation of the HPA axis. Persistent elevations of pro-inflammatory cytokines, particularly IL-6 and TNF-α, activate the enzyme IDO, diverting tryptophan metabolism away from serotonin synthesis towards the kynurenine pathway. This results in reduced serotonin availability and the accumulation of neuroactive metabolites, such as quinolinic acid, which promote glutamate-mediated excitotoxicity, oxidative stress, and altered dopamine neurotransmission. Together, these processes may contribute to depressive symptoms, emotional dysregulation, and cognitive impairment in patients with OSAS [11,38,39]. This process may contribute to depressive symptoms and emotional difficulties [38,39]. Ongoing sleep disruption and low oxygen levels can also make HPA-axis activity worse, adding to mood swings and depression in people who are vulnerable [4,38]. CPAP therapy may alleviate depressive symptoms by reducing the recurrent intermittent hypoxia and sleep fragmentation that drive systemic and neuroinflammatory responses in OSAS. By restoring upper airway patency during sleep, CPAP improves nocturnal oxygenation and normalizes sleep architecture, thereby reducing activation of pro-inflammatory pathways. Several studies have demonstrated reductions in circulating inflammatory biomarkers, particularly CRP, IL-6, and TNF-α, following sustained CPAP therapy. The reduction in these inflammatory mediators is thought to attenuate microglial activation, decrease activation of the kynurenine pathway, improve serotonin and dopamine neurotransmission, and partially restore HPA axis regulation, ultimately contributing to improvements in depressive symptoms [11,13,38,39]. Studies also indicate that using CPAP can help improve depressive symptoms by lowering CRP and IL-6 levels, which supports the link between mood disorders and inflammation [6,11,40,41]. Although CPAP has demonstrated beneficial effects on depressive symptoms and inflammatory biomarkers, the magnitude of improvement appears to depend on treatment adherence, baseline depression severity, and duration of therapy, highlighting the need for further long-term prospective studies.

Anxiety

It is also found that people who face repeated arousals with untreated OSAS have increased anxiety-related symptoms [11]. Experimental animal studies have demonstrated that SF and IH increase the expression of pro-inflammatory cytokines, including IL-1β, IL-6, and TNF-α, within the amygdala, thereby promoting neuroinflammatory signalling associated with anxiety-related behaviours [4,21]. These changes, along with altered GABAergic and glutamatergic neurotransmission and higher cortisol and catecholamine levels, lead to more anxiety-related behaviours such as fear [4,21]. In the central nervous system, neuroinflammation can disrupt stress-related regions and may contribute to autonomic dysregulation and heightened arousal, which are common in anxiety disorders [11,21,39]. Evidence also suggests that neuroinflammation in OSAS may affect brain circuits involved in emotional regulation, especially in the amygdala and limbic system. Repeated episodes of intermittent hypoxia and sleep fragmentation may overactivate the sympathetic nervous system, raise cortisol levels, and change neurotransmission, all linked to anxiety and hypervigilance [4,21,38]. Ongoing inflammation can keep both physical and psychological stress responses active, potentially contributing to persistent anxiety symptoms in untreated OSAS patients. For example, adults may have trouble concentrating at work, while children might perform worse in school [9,42]. Treatment of anxiety-related symptoms in patients with OSAS primarily focuses on correcting the underlying sleep-disordered breathing. CPAP, the first-line therapy for moderate-to-severe OSAS, reduces intermittent hypoxia and sleep fragmentation, thereby attenuating systemic and neuroinflammatory responses. Improvements in oxygenation and restoration of normal sleep architecture have been associated with reductions in inflammatory cytokines, sympathetic overactivity, and HPA axis dysregulation, mechanisms that are thought to contribute to improvements in anxiety symptoms. Several studies have also reported improvements in daytime alertness, emotional regulation, and overall quality of life following sustained CPAP therapy, although the magnitude of benefit may depend on treatment adherence, disease severity, and co-existing psychiatric conditions [11,13,38,40].

Cognitive Dysfunction

Sleep fragmentation and IH play pivotal roles in cognitive decline by inducing neuronal injury and oxidative stress [43,44]. Microglial activation and cytokine augmentation impair neurotransmission and spine integrity, leading to problems in working memory, attention, and executive functioning [9]. IH further precipitates the piling up of amyloid-β (Aβ) and tau hyperphosphorylation, commonly seen in the pathophysiology of Alzheimer’s disease (AD) and Parkinson’s disease (PD) [3,36,37,45]. Magnetic resonance imaging (MRI) indicates hippocampal atrophy that links up to impaired synaptic signalling in the hippocampus post-microglial activation [20,33,46]. In addition to hippocampal injury, neuroimaging studies have demonstrated widespread structural and functional brain abnormalities in patients with OSAS, particularly within the hippocampus, prefrontal cortex, anterior cingulate cortex, and other cortical regions involved in executive function, attention, and memory. Functional magnetic resonance imaging (fMRI) studies and comprehensive neurocognitive assessments have consistently identified impairments in verbal memory, sustained attention, working memory, executive function, and decision-making [14,15,46]. These issues are likely caused by ongoing inflammation and reduced oxygen supply to the brain [14,15,46]. While CPAP treatment has led to improvements in some cognitive areas, it is still unclear if long-term structural brain damage can be reversed [9,14]. Overall, current evidence suggests that these mechanisms may contribute to the association between OSAS and neurodegenerative processes.

Importantly, CPAP therapy, administered nightly during sleep to maintain upper airway patency and prevent recurrent airway collapse, has been associated with improvements in several cognitive domains, including verbal memory, attention, and executive function. These benefits are thought to result from improved nocturnal oxygenation, reduced intermittent hypoxia and sleep fragmentation, and attenuation of neuroinflammatory pathways. Nevertheless, although cognitive function may improve following sustained CPAP therapy, complete reversal of established structural brain abnormalities remains uncertain [9,11,14].

In summary, the outcomes of all mechanisms that interlink neuroinflammation make it clear that there is a complex network that links OSAS to various neuropsychiatric diseases. The growing evidence suggesting that CPAP reduces symptoms of depression and anxiety in OSAS patients has been widely recognized. However, important questions remain regarding patient selection, optimal treatment duration, and the long-term neurological benefits of CPAP therapy [9]. While we understand that CPAP therapy alleviates certain cognitive and inflammatory impairments, there is inadequate data on the effect of long-term CPAP usage on the modulation of neuroinflammatory biomarkers. Future research should explore CPAP use not only for slowing down the worsening symptoms but also to prevent the disease and offer other treatments besides CPAP to improve mood and cognition in such patients. With larger studies on CPAP use underway, this data remains underexplored. Eventually, establishing interventions to reduce and prevent neuroinflammation-mediated emotional and cognitive decline within a multidisciplinary framework encompassing psychiatry, sleep medicine, and neuroimmunology is essential.

Limitations of the existing literature

There is growing evidence that OSAS is associated with neuroinflammation and neuropsychiatric dysfunction; however, the existing literature has several important limitations. Much of the available evidence is derived from experimental animal models employing IH and SF, which cannot fully replicate the complex pathophysiology of human OSAS. Furthermore, considerable heterogeneity exists among studies with respect to diagnostic criteria, disease severity, exposure duration, neuroinflammatory biomarkers, imaging protocols, and neurocognitive assessments, limiting direct comparison of findings. Many investigations are observational or cross-sectional in design, making it difficult to establish causal relationships between OSAS, neuroinflammation, and neuropsychiatric outcomes. Although inflammatory biomarkers such as IL-6, TNF-α, and CRP are frequently investigated, there remains no standardized approach for their measurement or longitudinal monitoring. Likewise, the long-term effects of CPAP therapy on neuroinflammatory pathways, cognitive outcomes, and prevention of neurodegenerative disease remain incompletely understood [1,9]. Interpretation of the current evidence is further complicated by several important confounding factors. Obesity, increased body mass index (BMI), metabolic syndrome, hypertension, diabetes mellitus, and pre-existing cardiovascular disease are highly prevalent among individuals with OSAS and are independently associated with systemic inflammation, endothelial dysfunction, cognitive impairment, and mood disorders. Consequently, it remains challenging to determine the extent to which neuropsychiatric manifestations are directly attributable to OSAS rather than to these co-existing conditions. Although many studies attempt to adjust for these variables statistically, residual confounding cannot be excluded and may partially explain the variability observed across published findings [5,8,9,27]. As summarised in Table 1, the current evidence comprises a heterogeneous combination of experimental studies, observational clinical studies, systematic reviews, and meta-analyses, highlighting both the breadth of available research and the continuing need for high-quality longitudinal investigations. Furthermore, as this review follows a narrative methodology, study selection and evidence synthesis may be subject to selection bias, and no formal quality or risk-of-bias assessment was performed. Therefore, the conclusions should be interpreted within the context of the available evidence. 

This narrative review has several strengths. It integrates current evidence from experimental, translational, and clinical studies to provide a comprehensive overview of the neuroinflammatory mechanisms linking OSAS with depression, anxiety, cognitive dysfunction, and neurodegenerative disease. By synthesizing findings across multiple disciplines, including sleep medicine, neuroscience, psychiatry, and neuroimmunology, the review highlights emerging mechanistic pathways such as microglial activation, blood-brain barrier dysfunction, glymphatic impairment, and the potential therapeutic role of CPAP.

Nevertheless, several limitations should be acknowledged. As a narrative review, the study did not employ a formal systematic review methodology or quantitative meta-analysis, and study selection may therefore be subject to selection bias. Although the literature search was comprehensive, differences in study design, patient populations, outcome measures, and biomarker assessment limited direct comparison between studies. Consequently, the conclusions presented should be interpreted within the context of the available evidence, and further large-scale longitudinal studies and randomized controlled trials are needed to clarify causal mechanisms and identify effective therapeutic targets.

Overall, the available evidence comprises experimental studies, observational clinical studies, systematic reviews, and meta-analyses and consistently supports an association between OSAS, neuroinflammation, and neuropsychiatric dysfunction. However, relatively few large prospective longitudinal human studies are currently available, limiting definitive conclusions regarding causality. These findings highlight the need for well-designed longitudinal studies and randomized controlled trials to further clarify disease mechanisms and optimize therapeutic strategies.

Future research directions

The findings of this review have several important clinical and research implications. By integrating current evidence on neuroinflammatory mechanisms, this review highlights the importance of recognizing obstructive sleep apnoea syndrome (OSAS) as a multisystem disorder with potential neurological and psychiatric consequences rather than solely a sleep-related breathing disorder. Improved understanding of the roles of intermittent hypoxia, sleep fragmentation, microglial activation, and glymphatic dysfunction may facilitate earlier identification of patients at increased risk of cognitive impairment, depression, anxiety, and neurodegenerative disease, while supporting the development of targeted therapeutic strategies beyond conventional CPAP therapy.

Future research should focus on better understanding how OSAS is linked to neuroinflammation and neuropsychiatric disorders. Larger, prospective longitudinal studies incorporating standardized assessment of neuroinflammatory biomarkers are needed to clarify causal mechanisms, validate biomarker profiles, and identify reliable biomarkers and predictors of cognitive and neuropsychiatric disease progression in patients with OSAS [9,12,18,34]. Advanced neuroimaging techniques, including fMRI and molecular imaging, may further elucidate how intermittent hypoxia and sleep fragmentation alter the structure and function of the central nervous system [9,46]. Researchers should also investigate targeted anti-inflammatory and neuroprotective therapies alongside standard CPAP treatment to determine whether modulation of neuroinflammatory pathways can reduce the long-term risk of cognitive decline and psychiatric morbidity [21,34]. As precision medicine and biomarker-guided therapeutic approaches continue to evolve, early identification of patients at increased risk of neurodegenerative progression may enable more timely intervention [18,19]. Finally, large multicentre prospective studies evaluating long-term CPAP adherence, neuroinflammatory biomarkers, and neurological outcomes are required to establish evidence-based treatment strategies and optimize long-term patient care. Future studies should also prioritize multicentre collaborative research involving diverse patient populations to improve the generalizability of biomarker-based risk stratification and therapeutic interventions.

## Conclusions

Obstructive sleep apnea syndrome (OSAS) is increasingly recognized as a condition with implications beyond sleep-related breathing disturbances. Accumulating evidence suggests that OSAS is associated with neuropsychiatric disorders through multiple neuroinflammatory mechanisms. Intermittent hypoxia and sleep fragmentation are thought to contribute to oxidative stress, cytokine-mediated neuronal injury, microglial activation, and disruption of the blood-brain barrier. These pathophysiological changes may negatively affect mood, cognition, and overall neurological function. Collectively, experimental and clinical studies support an association between OSAS and depression, anxiety, cognitive impairment, and neurodegenerative disorders, emphasizing the importance of early diagnosis and intervention.

Continuous positive airway pressure (CPAP) therapy has been shown to improve certain cognitive and emotional symptoms in individuals with OSAS; however, its long-term effects on neuroinflammation and neuroprotection remain unclear. Current evidence indicates that neuroinflammation serves as a critical mediator between OSAS and neuropsychiatric disorders. Although the available evidence is compelling, much of it remains associative and is derived from heterogeneous experimental and clinical studies, highlighting the need for cautious interpretation and further validation. Further longitudinal, biomarker-driven, and multi-centre studies are required to elucidate causal relationships, monitor long-term neurological outcomes, and identify novel therapeutic strategies. Advancing our understanding of these mechanisms may facilitate earlier diagnosis, improved risk stratification, and the development of targeted therapeutic strategies to mitigate cognitive and emotional deterioration in individuals with OSAS.
